# Sleep Duration in Mouse Models of Neurodevelopmental Disorders

**DOI:** 10.3390/brainsci11010031

**Published:** 2020-12-30

**Authors:** Rachel Michelle Saré, Abigail Lemons, Alex Song, Carolyn Beebe Smith

**Affiliations:** Department of Health and Human Services, National Institutes of Health, National Institute of Mental Health, Section on Neuroadaptation and Protein Metabolism, Bethesda, MD 20814, USA; Rachel.Sare@nih.gov (R.M.S.); abigaillemons7@gmail.com (A.L.); alexsong2014@u.northwestern.edu (A.S.)

**Keywords:** neurodevelopmental disorders, sleep duration, Phelan–McDermid syndrome, Shank3, oxytocin receptor, tuberous sclerosis

## Abstract

Sleep abnormalities are common in patients with neurodevelopmental disorders, and it is thought that deficits in sleep may contribute to the unfolding of symptoms in these disorders. Appreciating sleep abnormalities in neurodevelopmental disorders could be important for designing a treatment for these disorders. We studied sleep duration in three mouse models by means of home-cage monitoring: *Tsc2*^+/−^ (tuberous sclerosis complex), oxytocin receptor (*Oxtr*) knockout (KO) (autism spectrum disorders), and *Shank3 ^e4-9^* KO (Phelan–McDermid syndrome). We studied both male and female mice, and data were analyzed to examine effects of both genotype and sex. In general, we found that female mice slept less than males regardless of genotype or phase. We did not find any differences in sleep duration in either *Tsc2*^+/−^ or *Oxtr* KO mice, compared to controls. In *Shank3 ^e4-9^* KO mice, we found a statistically significant genotype x phase interaction (*p* = 0.002) with a trend that *Shank3* *^e4-9^* KO mice regardless of sex slept more than control mice in the active phase. Our results have implications for the management of patients with Phelan–McDermid syndrome.

## 1. Introduction

Sleep abnormalities are prevalent in patients with neurodevelopmental disorders. In particular, 50–85% of patients with autism spectrum disorders (ASD) are reported to have some sort of sleep disturbance, most commonly reduced total sleep duration [1]. We hypothesized that sleep reduction may play a role in the unfolding of these disorders [1] and determined that it is an important phenotype to investigate. Patient studies of neurodevelopmental disorders can be problematic because many factors can affect sleep, including sleep hygiene, medications, and other environmental influences, and these factors are difficult to control in clinical studies.

Mouse models offer an alternative means to investigate sleep abnormalities in ASD and their molecular underpinnings. Published reports indicate sleep abnormalities in several mouse models of neurodevelopmental disorders, including Angelman syndrome [2], Down syndrome [3], fragile X syndrome [4,5], and Rett syndrome [6]. Several mouse models of ASD have also been reported to have sleep abnormalities, including 16p11.2 deletion [7], BALBC [8], *Gabrb3* KO [9], *neuroligin-1* KO [10], and *neuroligin-3* KO [11] mice.

Tuberous sclerosis complex (TSC) is characterized by a mutation in either the *TSC1* or *TSC2* genes [12]; approximately 50% of patients with TSC have ASD [13]. A few reports of patients with TSC suggest the prevalence of sleep abnormalities in 30–90% of patients [1]. Circadian rhythm abnormalities have been reported in a mouse model of TSC [14], though sleep behavior per se has not been examined. Oxytocin is an important hormone involved in social bonding, and *Oxtr* KO mice have been shown to exhibit impaired social interactions and impaired social communication [15]. Sleep behavior has not been examined in this model. Phelan–McDermid syndrome is characterized by deletion of the chromosomal region 22q13, which contains the SHANK3 gene. Point mutations in SHANK3 can cause many of the features of Phelan–McDermid syndrome, leading some to believe that it is causally involved in the disorder [16]. In patients with Phelan–McDermid syndrome, 25% have ASD and 90% may have sleep problems [17]. A recent study showed that *Shank3^∆C^* KO mice had decreased sleep duration in the active phase, difficulty with sleep rebound, and abnormal circadian rhythm [18].

We sought to further characterize sleep in additional mouse models of neurodevelopmental disorders. Here, we used home-cage monitoring to study sleep in *Tsc2^+/−^*, *Oxtr* KO, and *Shank3 ^e4-9^* KO mice. We found that sleep duration is not statistically significantly different in either *Tsc2^+/−^* or *Oxtr* KO mice compared to their respective controls. In *Shank3 ^e4-9^* KO mice, we found a statistically significant genotype x phase interaction indicating genotype-dependent and sex-independent differences in sleep duration in the active phase. Our results have implications for the management of patients with Phelan–McDermid syndrome. 

## 2. Materials and Methods

### 2.1. Animals

All animal procedures were conducted in accordance with the National Institutes of Health Guidelines on the Care and Use of Animals and were approved by the National Institute of Mental Health Animal Care and Use Committee (LCM-07). Mice were kept on a standard 12:12 light:dark cycle with lights on at 6:00 AM. Mice had access to food and water *ad libitum* with standard housing conditions in a central facility. Animals were weaned at 21 days of age. DNA was collected from either tail or ear clippings for genotyping.

### 2.2. TSC

*Tsc2^+/−^* and WT breeding pairs on a C57BL/6J background were a gift from Mark Bear (MIT, Boston, MA, USA). These animals are commercially available from Jackson Labs (Jackson Labs: B6; 129S4-Tsc2tm1Djk/J Stock No: 004686). Mice were genotyped as described by Jackson Labs (http://www.jax.org/strain/004686). *Tsc2^+/−^* mice and WT littermates were studied. 

### 2.3. Oxtr KO

*Oxtr^+/−^* breeding pairs on a C57BL/6J background were a gift from Scott Young (NIMH, Bethesda, MD, USA) and were genotyped as previously described [19]. *Oxtr* KO, *Oxtr^+/−^*, and WT littermates were studied.

### 2.4. Shank3 ^e4-9^ KO

*Shank3 ^e4-9+/−^* breeding pairs on a C57BL/6J background were purchased from Jackson Labs (Bar Harbor, ME, USA) (B6.129S7-Shank3tm1Yhj/J Stock No: 017442) and were genotyped as described by Jackson Labs (https://www.jax.org/strain/017442). *Shank3 ^e4-9^* KO, and *Shank3 ^e4-9+/−^*, and WT littermates were studied.

### 2.5. Home-Cage Assessment of Sleep Duration

At 60–77 days of age, we assessed sleep duration by means of the home-cage activity monitoring system (Comprehensive Laboratory Animal Monitoring System (CLAMS)) (Columbus Instruments, Columbus, OH, USA) as previously described [20]. Briefly, mice were singly housed in a standard mouse cage for the duration of the sleep assessment. Each cage was placed in a rectangular arena surrounded by infrared emitters and sensors. Photobeams were placed 0.5 inches apart in both the x and y planes to generate activity data on a high-resolution grid. The Oxymax program (Columbus Instruments) was used to detect activity in 10 s epochs. If no activity was recorded in four consecutive epochs (40 s), the animal was considered asleep. This measure has been previously validated against electroencephalogram-defined sleep in C57BL/6J mice [21]. Sleep was analyzed for 72 consecutive hours. To eliminate the effects of habituation, the first 24 h were not analyzed [4].

### 2.6. Statistical Analysis

All data presented as mean ± standard error of the mean (SEM) were analyzed by means of repeated measures ANOVA with phase (active, inactive) as a within subject variable and genotype and sex as between subjects variables. ANOVA results are presented in Table 1. When appropriate, Bonferroni corrected post-hoc *t*-tests were performed. *p*-values ≤ 0.05 were considered statistically significant and are denoted with a “*”.

## 3. Results

### 3.1. Sleep Duration in a Mouse Model of TSC

**Figure 1:**
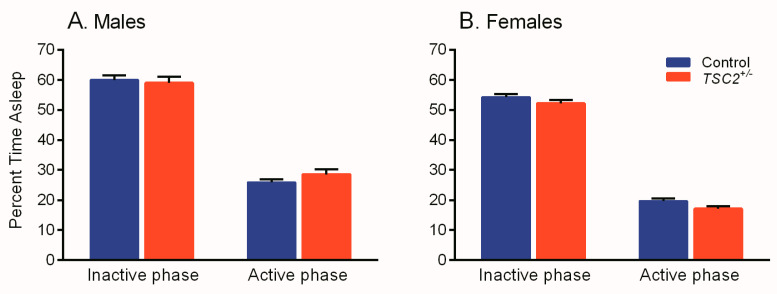
Sleep duration in *Tsc2^+/−^* and control male (**A**) and female (**B**) mice. We found statistically significant main effects of phase and sex, but no statistically significant interaction or main effects of genotype (Table 1). Mice slept less in the active phase than in the inactive phase (*p* < 0.001). Females slept less than male mice (*p* < 0.001). Bars represent means ± SEM for 22 control male, 22 *Tsc2^+/−^* male, 23 control female, and 21 *Tsc2^+/−^* female mice.

We analyzed sleep duration in male and female adult *Tsc2^+/−^* and control mice (raw data in Supplemental Table S1) and found statistically significant main effects of phase (*p* < 0.001) and sex (*p* < 0.001) but no interactions or main effects of genotype (Table 1). These data show that animals regardless of genotype or sex slept more in the inactive phase (light) than the active phase (dark) (Figure 1). Additionally, regardless of phase or genotype, females slept less than males (Figure 1).

### 3.2. Sleep Duration in Oxtr KO Mice

**Figure 2:**
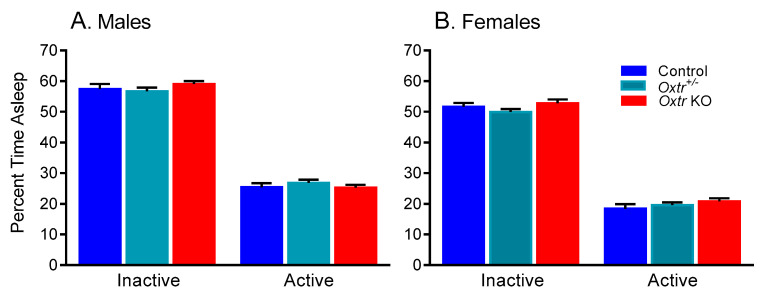
Sleep duration in oxytocin receptor (*Oxtr*) *Oxtr* knockout (KO), *Oxtr^+/^^−^*, and control male (**A**) and female (**B**) mice. We found statistically significant main effects of phase and sex, but no statistically significant interactions or main effects of genotype (Table 1). Mice slept less in the active phase compared to the inactive phase (*p* < 0.001). Females slept less than males (*p* < 0.001). Bars represent means ± SEM for 23 control male, 21 *Oxtr^+/−^* male, 22 KO male, 14 control female, 31 *Oxtr^+/−^* female, and 21 KO female mice.

In male and female adult control, heterozygous, and *Oxtr* KO mice (raw data in Supplemental Table S2, we found statistically significant main effects of phase (*p* < 0.001) and sex (*p* < 0.001) but no statistically significant interactions or main effects of genotype (Table 1). These data show that animals, regardless of genotype or sex, slept more in the inactive phase than the active phase (Figure 2). Additionally, regardless of phase or genotype, females slept less than males (Figure 2).

### 3.3. Sleep Duration in Shank3 ^e4-9^ Mouse Model of Phelan–McDermid Syndrome

**Figure 3:**
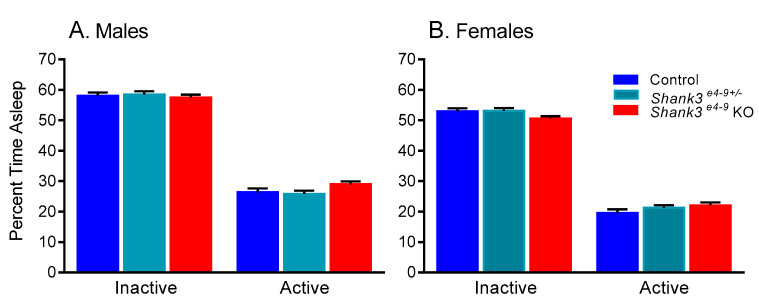
Sleep duration in *Shank3 ^e4-9^* KO, *Shank3 ^e4-9+/^^−^*, and control male (**A**) and female (**B**) mice. We found a statistically significant genotype x phase (*p* = 0.002) interaction (Table 1). Post-hoc tests showed that KO mice tended to sleep more in that active phase compared to control mice (*p* = 0.059). We also found statistically significant main effects of phase and sex (Table 1). Mice slept less in the active phase than in the inactive phase (*p* < 0.001). Female mice slept less than male mice (*p* < 0.001). Bars represent means ± SEM for 20 control male, 31 *Shank3 ^e4-9+/−^* male, 22 KO male, 23 control female, 25 *Shank3 ^e4-9+/−^* female, and 22 KO female mice.

In male and female adult control, heterozygous, and *Shank3 ^e4-9^* KO mice (raw data in Supplemental Table S3), we found a statistically significant genotype x phase interaction (*p* = 0.002) (Table 1). Regardless of sex, *Shank3 ^e4-9^* KO mice tended to have increased sleep (11%) compared to control mice in the active phase (*p* = 0.059), but sleep duration in the inactive phase was similar for all three genotypes (Figure 3). Main effects of phase (*p* < 0.001) and sex (*p* < 0.001) were also statistically significant (Table 1). These data show that animals regardless of genotype or sex slept more in the inactive phase than the active phase (Figure 3), and regardless of phase or genotype, females slept less than males (Figure 3).

## 4. Discussion

Sleep duration in genetic mouse models of TSC, Phelan–McDermid syndrome, and ASD was affected only minimally. In all models, we confirmed a large difference in sleep duration between the active and inactive phases. We also consistently found that there was a large sex difference, such that female mice slept significantly less than male mice. Effects on sleep duration due to genotype were not striking. The only model in which a difference in sleep duration was noted was the *Shank3 ^e4-9^* KO model in which we found a significant genotype x phase interaction and a trend toward increased sleep duration in the active phase.

The methodology we used in this study is simple to execute and allows for the study of large groups of animals useful for statistical comparisons. No surgical intervention is required, so confounds due to anesthesia and surgery are avoided. Home-cage monitoring, however, can only measure sleep duration and may miss more subtle changes in sleep patterns such as effects on stages of sleep and sleep spindles. It is hypothesized that rapid eye movemnt sleep abnormalities are prevalent in patients with ASD [22] and that patients with ASD have decreased sleep spindles [23]. Therefore, it may be that subtle sleep abnormalities may still play a role in neurodevelopmental disorders and/or ASD.

Sleep is regulated by two processes: circadian rhythm and sleep homeostasis [24]. Abnormalities in circadian rhythm and associated genes are hypothesized to be involved in ASD [25]. Another possibility is that sleep abnormalities in neurodevelopmental disorders may result from reduced response to sleep homeostatic demands. It may be that sleep in optimal environments is unaltered, but if sleep is disrupted due to, for example, noise, changes in temperature, or a novel environment, patients may be unable to rebound and recover sleep loss. Examining the homeostatic response to sleep loss in mouse models of neurodevelopmental disorders may be a useful indicator of such abnormalities.

We studied mouse models of three neurodevelopmental disorders: TSC, Phelan–McDermid syndrome, and ASD. Although we did not detect significant differences in sleep duration in *Tsc2^+/−^* mice, circadian rhythm abnormalities have been observed. In constant darkness, *Tsc2^+/−^* mice had a shorter free-running period than control mice [14]. In *Oxtr* KO mice, we did not detect differences in sleep duration, but the Oxtr has been implicated in circadian rhythm regulation. Studies in hamsters demonstrate that injection of an Oxtr agonist or antagonist inhibited light-induced phase advances or light-induced phase delays, respectively [26]. Oxytocin likely plays a role in the phase delay experienced by light in the early night.

Our findings in *Shank3 ^e4-9^* KO mice showing a significant genotype x phase interaction suggest increased sleep in the active phase compared to control mice. Our results contrast with a recent EEG study of *Shank3^∆C^* KO mice in which reduced sleep duration in the active phase was reported [18]. The affected isoform of Shank3 differed in our study, suggesting that Shank3 may be involved in the regulation of sleep but in a complex manner. Our results are in agreement with a recent study by Angelakos et al., who reported hypoactivity in the active phase of *Shank3 ^e13-16^* KO mice [27]. Interestingly, they also report hypoactivity in the active phase in three other mouse models of neurodevelopmental disorders: *Cntnap2^−/−^*, *Pcdh10^+/−^*, and *Fmr1* KO mice [27].

One of our robust findings is that females consistently slept less than male mice regardless of genotype (about 10% difference in the active phase and 25% difference in the inactive phase). It is important to note that we did not control for the estrous cycle, and this could be a potential confound of the results. This sex difference in sleep duration has been noted previously in mouse studies [28,29]. In human populations, women are not reported to have reduced sleep duration compared to men; however, they do have increased sleep onset latencies and have a higher incidence of insomnia compared to men [30]. This may contribute to a higher susceptibility to mood disorders and to Alzheimer’s disease in females [31,32]. Our studies highlight the importance of separating males and females in studies of sleep.

In summary, reduced sleep duration, as determined by home-cage monitoring, was not seen in any of the three mouse models of neurodevelopmental disorders examined in our study. It could still be that more subtle differences in sleep characteristics may occur in neurodevelopmental disorders, so future studies should address potential differences in REM sleep as well as sleep homeostasis. 

## Figures and Tables

**Table 1:** Results of repeated measures ANOVA with corresponding F-values and *p*-values.

Model	Interaction	Main Effect	F(df, Error) Value	*p*-Value
*Tsc2^+/−^*				
	Sex × genotype × phase		F(1, 84) = 1.325	0.253
	Sex × phase		F(1, 84) = 2.361	0.128
	Genotype × phase		F(1, 84) = 1.210	0.274
	Sex × genotype		F(1, 84) = 1.921	0.169
		Sex	F(1, 84) = 49.048	<0.001 *
		Genotype	F(1, 84) = 0.312	0.578
		Phase	F(1, 84) = 1806.221	<0.001 *
*Oxtr* KO				
	Sex × genotype × phase		F(2, 126) = 1.022	0.363
	Sex × phase		F(1, 126) =0.005	0.945
	Genotype × phase		F(2, 126) = 0.983	0.377
	Sex × genotype		F(2, 126) = 0.396	0.674
		Sex	F(1, 126) = 63.486	<0.001 *
		Genotype	F(2, 126) = 0.987	0.375
		Phase	F(1, 126) = 2481.879	<0.001 *
*Shank3 ^e4-9^* KO				
	Sex × genotype × phase		F(2, 137) = 0.537	0.586
	Sex × phase		F(1, 137) =0.125	0.725
	Genotype × phase		F(2, 137) = 6.480	0.002 *
	Sex × genotype		F(2, 137) = 0.708	0.494
		Sex	F(1, 137) = 74.197	<0.001 *
		Genotype	F(2, 137) = 0.230	0.795
		Phase	F(1, 137) = 3771.461	<0.001 *

*p*-values ≤ 0.05 were considered statistically significant and are denoted with a “*”.

## Data Availability

Data is contained in a supplemental file as part of this article.

## Supplementary Materials

The following are available online at https://www.mdpi.com/2076-3425/11/1/31/s1, Table S1: Raw sleep duration data for *TSC^+/^*^−^ mice and controls, Table S2: Raw sleep duration data for *Oxtr* KO, *Oxtr^+/^*^−^, and controls, Table S3: Raw sleep duration data for *Shank3 ^e4-9^* KO, *Shank3 ^e4-9^* ±, and controls.
